# Correction: The effects of probabilistic context inference on motor adaptation

**DOI:** 10.1371/journal.pone.0331934

**Published:** 2025-09-08

**Authors:** Dario Cuevas Rivera, Stefan Kiebel

Fig 1 is a duplicate of Fig 2. Please see the correct Fig 1 here.

**Fig 1:**
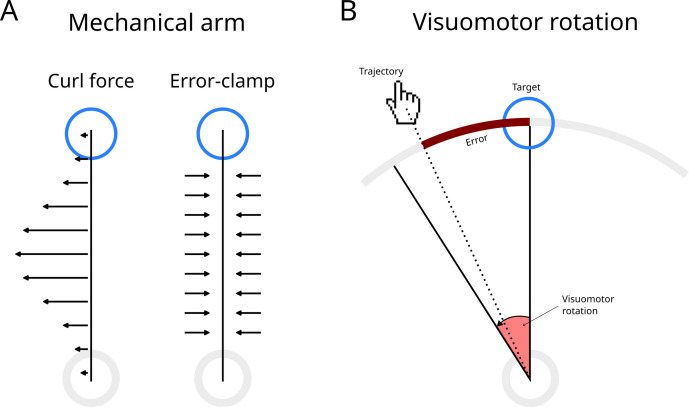
Mechanical arm and visuomotor rotation experiments. Grey and blue circles represent the starting point and the target, respectively. Targets are placed on a circumference in both experimental paradigms. (A) Mechanical arm experiment. During adaptation trials, the force exerted by the arm on the handle pushes the hand away from the straight line (i.e., the force is perpendicular to the direction of movement). In error-clamp trials, the mechanical arm creates resistance against movements perpendicular to the straight line to the target. (B) Visuomotor rotation experiment. The correspondence between hand movements and the cursor on the screen (hand icon) is rotated (visuomotor rotation). The error (brown line) is measured as the arc between the target and the place at which the trajectory (dotted line) crossed the circumference.
