# Supplementary material for: The effects of probabilistic context inference on motor adaptation
Source: PLoS One. 2023 Jul 3;18(7):e0286749. doi: 10.1371/journal.pone.0286749 (PMC10317241; doi:10.1371/journal.pone.0286749)
Supplement: Attachment — Submitted filename: PLOS_ONE_review.pdf [file pone.0286749.s001.pdf]

The effects of probabilistic context inference on motor adaptation

Reviewer summary: Recently, computational models and Bayesian for motor adaptation have been introduced to demonstrate the effects of context inference on learning rates in various experiments. Cuevas Rivera, Darío and Kiebel built a simplified version of the recently introduced COIN model to demonstrate that the effects of context inference on motor adaptation and control extend even further than previously demonstrated. They used this model to simulate previous motor adaptation experiments and demonstrated that context inference, and how it is affected by the presence and reliability of feedback, affect a variety of behavioral phenomena which had previously required multiple hypothesized mechanisms. The authors suggest that the reliability of direct contextual information, as well as noisy sensory feedback, which is common in many experiments, cause measurable changes in switching-task behavior and action selection that are directly related to probabilistic context inference.

I think the manuscript in its current state is a good starting point. The overall clarity of the writing needs improvement. However, I feel as though the authors lack a clear understanding of the behavioural phenomena they are attempting to model. Savings is not the absence of learning upon re-exposure to the same previously experienced perturbation, but faster relearning. Error clamps are not a zero force manipulation as in baseline trials. There is a matching of the force generated by participants and visually displayed to participants as zero error. Any previously learned perturbations will decay at this block of trials, rather than a learning of zero error.

Minor issues:

Lines 33-35 could add reference: Howard, I. S., Wolpert, D. M., & Franklin, D. W. (2013). The effect of contextual cues on the encoding of motor memories. Journal of neurophysiology, 109(10), 2632-2644.

Lines 40-41 add “upon re-exposure to the same perturbation”

The ability to recall previously learned skills **upon re-exposure to the same perturbation**, called savings;

Line 47 - models don’t do the adapting. “Whereby after each iteration, the change in motor commands is related to the measure of error.”

Line 47 The most **well-**studied **models** are …”

Lines 49-50 from “but Bayesian accounts have also been presented, providing an alternative explanation for savings and quick de-adaptation in the form of switching between forward models” to “**while** Bayesian **models have been shown to provide** an alternative explanation for savings and quick de-adaptation in the form of switching between forward models.”

Lines 61-63, “Heald et al. (2021) formalized context inference as a process that

operates independently from motor learning, but is informed by it, establishing a loop whereby context inference also informs motor learning.”

but is informed by it - this is unclear

Line 66 - in this work **we** show

Line 70 - remove “so-called”

Line 104 - add “s” to component

Figure 1. How are motor commands “observable”?

Lines 137-138. The robot motors exert a force on the handle of the robot, held by the participant. It does not exert force on the participant’s hand.

Lines 154-157. More clearly stated as the output of the simulated data qualitatively matches the behavioural data observed in ….. <list of studies>

Line 160 add “s” to provide

Line 176-177 “in which the mechanical arm forces the participant to make straight-line movements” This is incorrect. The robot is programmed with spring and damping coefficients to match the force exerted by the participant and make it appear as though they are making a perfect trajectory from start position to target (i.e., the elimination of the visual error).

Lines 182-183 “With repeated contexts (e.g. Oh & Schweighofer, 2019), an experiment can be described as O1. A1.O2”. This is unclear.

Line 186 “without having to re-learn it.” They are learning in the second session of A, it is just that they are learning at a faster rate.

Line 192. In the baseline condition there is nothing to learn (i.e., there is not perturbation). In this case there is a washout or decay of the learning of A.

Line 195 and 199 replace “are” with “is”

Did the simulated data represent the same number of subjects reported in the compared studies? This is unclear in the reading.

Can the authors explain why their model predicts no savings upon the 4th presentation of the perturbation when the behavioural data to the right clearly shows savings. (From figure 2C). The learning rate of the model clearly lags behind the data.

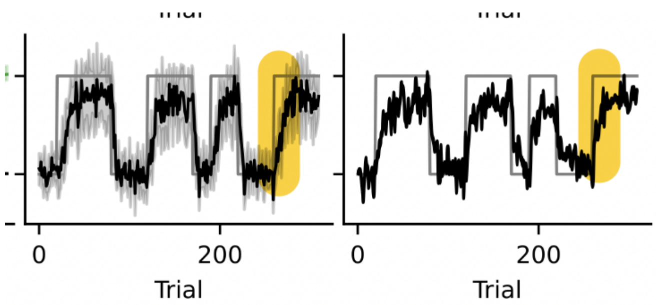

Line 249 replace “do” with “does”

Line 348, had begun or began

Lines 407 - 413 There is no mention of the decay happening in error clamps. “Instead, participants slowly reduce their adaptation, often displaying spontaneous recovery” FIrst you get rebound and then you get decay … this statement is misleading.

413-417 Participants are often unaware of the presence of error clamps. How can the authors account for this finding in relation to the literature? I.e., Scheidt, R. A., Reinkensmeyer, D. J., Conditt, M. A., Rymer, W. Z., & Mussa-Ivaldi, F. A. (2000). Persistence of motor adaptation during constrained, multi-joint, arm movements. Journal of neurophysiology, 84(2), 853-862.. Experiments often interleave error clamp trials within the adaptation blocks. Can your sCOIN model account for the changes in behaviour during these trials, given that participants are often unaware they even occur?

Major issues:

Why are the simulations shown with SD and compared to participant data often using SEM?

Introduction lacks a clear telling of the COIN model in its original form and an explanation for why it cannot account for various phenomena (i.e., which phenomena are not accounted for by the COIN model). This would open the discussion better to why changes made to the model would account for them.

The interpretation of error clamps suggests a lack of understanding of what they are and how they are used in the motor adaptation, force field literature. See comments related to lines 413-417.

Overall there lacks a clear and direct comparison (statistically between simulated outcomes and behavioural data). The comparisons are of a more qualitative nature. The authors could provide a regression metric.

It would also be interesting to see not only the simulated data from the sCOIN model but also the COIN model to demonstrate where the COIN model fails to predict the data while the sCOIN improves the prediction.

Rather than separate presentations of simulated model output and experimental data, these two learning curves could be overlaid in contrasting colors to highlight their similarities or differences.
