# Supplementary material for: The effects of probabilistic context inference on motor adaptation
Source: PLoS One. 2023 Jul 3;18(7):e0286749. doi: 10.1371/journal.pone.0286749 (PMC10317241; doi:10.1371/journal.pone.0286749)
Supplement: Attachment — Submitted filename: answer_to_reviewers.pdf [file pone.0286749.s002.pdf]

Response to reviewers

Reviewer 1

We appreciate the reviewer's time and very helpful comments.
In our responses below, all line numbers refer to the new manuscript; note that the reviewers' comments refer to the lines of the original submission and our line numbers refer to the revised version.

Figure 2: What does the gray region around the black line in the third and fourth panels represent? Why there is no black line in the third column of 2A as opposed to the other experiments (2B and 2C).

Yes, this was unclear. To address this comment and to keep consistency with experimental results, we have changed these plots. The plots for our simulations are now mean +- SEM. We have added this information to the figures' captions, including Figure 3 (previously figure 2).

Figure 1: In the left figure of panel B, if the context changed at trial 20, It is not clear how the inferred state (force) does not change even in the high certainty and low noise setup? It may be due to the fact that I do not understand the experiment setup. Please clarify.

During the first 20 trials, context A was inferred and its internal model updated, as shown in Figure 2B (left). However, at trial 20 the context changes, which is inferred by the model (with a lag of 1 trial in the case of high cue uncertainty), which means that the inferred state for context A is no longer updated. Despite this, the actions selected by the model do change after trial 20, switching to a zero-force adaptation consistent with the baseline context. The one exception is in the case of high cue uncertainty and high observation noise (in green), in which case context inference is not a hundred percent certain, which causes context A to still be updated, albeit more slowly than before.
We have added a note to this effect in the manuscript, lines 220-224.

It is not clear to me (Despite the reasons given in the paragraph starting in line 523) why the authors did not try to fit the sCoin model parameters to real subjects data instead of arbitrarily/empirically selecting them for the simulation.

We thank the reviewer for this question. There were three reasons why we performed qualitative comparisons with selected parameter values. Firstly, the key point of the manuscript is to show that the context-inference approach shown here provides a unifying explanation for a host of phenomena discussed in this manuscript, as well as in Heald (2021) and Oh (2019). Using the current approach shows that the adaptability of the model supports our conclusions.
Secondly, our intention was to show that the concept of context inference itself is responsible for the behavioral phenomena, and not a specific mathematical implementation of the model, or the fitting process. Although showing a fitting process would be clearly useful for future experimental applications, the fitting itself would not have enhanced the conclusions reached in this manuscript.
Thirdly, we believe that it would be best to design new experiments specifically to test the predictions laid out in the Discussion section and design the fitting procedure accordingly. Under these circumstances, a qualitative comparison between the behavior predicted by the model and that of participants can be done directly. We will do this in future work.
We have added this discussion to the Discussion section of the manuscript (subsection called "Future work", lines 571-575).

I am missing here the link between the observed data (both real and simulated) and the model. Which component/s of the model are responsible for the main observed phenomena? It would help the reader better understand the model workings and appreciate its ability to exhibit those behaviors (similar to how it was done later on in the paragraph starting from line 274.)

Following the reviewer's comment, we rephrased that paragraph (and the following one) to make a direct link to model components. The new paragraph is in lines 337-340.

Line 136: provide more information on the "generic experiment". It would be easier to understand how contextual cues and sensory feedback are provided and noised. (Instead of just referring the reader to Davidson and Wolpert (2004))

We added a brief description of the experiment in this section, which can be seen in lines 205-211 and 214-217.

Figure 3A: In the Davidson and Wolpert (2004) paper, as far as I understand the post switch experiment length is 40 trials not 60. Can you elaborate on the difference? (below)

We thank the reviewer for pointing this out. We mistakenly let the simulation run for 20 extra trials. We have now corrected this (see Fig. 4a), as well as the x-axis labels on the experimental data. The results did not change qualitatively.

In Figure 4C group 1.2, it is not clear why the posterior for the baseline context starts from 0.5 while in the other groups it starts with 1.

Yes, this was inconsistent. Group 1.2 was the only one to start with baseline trials in the experiment, and the rationale was that we wanted to avoid giving the model the true context (in the form of priors over contexts) at the beginning of the simulations. This led to completely uninformative priors (i.e. 50/50). However, as this difference did not impact results at all, we have now removed this distinction and started group 1.2 with the same priors as the others, to improve the clarity (see Fig. 5C).

TODO Line 338: It would help if you explicitly explain what parts of the sCOIN model cause the increase in the probability of the baseline context in EC trials.

At first, right at the beginning of the EC trials, it is not that the baseline context is increasing its probability, but rather that the previously inferred context is no longer producing predictions that are matched and is therefore losing in probability. This is because the internal model for this context predicts a spread of errors (as $p(y_t|x_t) = N(y_t - \hat{y}, \hat{s}igma)$, from eqn. 11, where $\hat{s}igma$ depends on the context), but observes all errors as zero because of the error clamp. As the EC trials continue, action selection is made through a mix of both contexts (eqn. 6), and the continued observation

of zero error starts to favor the baseline context because it has a higher probability density of observing an error of exactly zero (since it has been practiced more by the participant/model and the standard deviation $\hat{s}igma$ is smaller for baseline than for adaptation contexts during the experiment). We have added this to the manuscript (lines 439-442), as well as more detail to the description of the sCOIN model in the Methods to better explain this phenomenon (equations 9-10, and lines 654-663).

Line 452: Base this prediction on the mechanisms of the model, beyond the explanation that it is allowed by the process of context inference.

Following the reviewer's comment, we now clarify which components of the model make the prediction in that section. The new paragraph is in lines 524-535.

Line 542: use consistent notation: Either δ or d; either c or ζ.

We apologize for these oversights. We have corrected the inconsistencies identified by the reviewer, as well as other (minor) notational errors in the Methods section.

Code: It is not clear how to run the code in the repository. Please specify the entry point/s and add other needed information about how to run the project to reproduce the simulation results presented in the paper.

We apologize for this. We prepared a simplified version of the full codebase used for the simulations, but it was unfortunately not at the forefront of the linked repository.
To correct this, we have created a new repository that contains only the necessary code to run the simulations. Additionally, we included detailed instructions in the README file to run the code, as well as the required packages to do so.

Typos and small corrections

The following typos and other erroneous details were corrected:

1. Line 66: ungrammatical "In this work show that"
2. Line 125: typo "alread-learned".

3. Figure 1: In panel A mention that this is the Schematic representation of the sCOIN model (rather than COIN Model).
4. Line 218: which one is the center panel in figure 2A? There are 4...
5. Line 250: typo “ever” instead of “every”.
6. Line 289: typo "O" instead of "0".
7. Figure 2: No blue lines in the figure. Do you mean orange?
8. Figure 3: Use the model name consistently throughout the paper, either sCOIN or SCOIN.
9. Figure 3 caption: in the 4’th line, Group -A instead of group A.
10. Line 542: use consistent notation: Either δ or d; either c or ζ.

Reviewer 2

We appreciate the reviewer’s time and very helpful comments.
In our responses below, all line numbers refer to the new manuscript; note that the reviewers’ comments refer to the lines of the original submission and our line numbers refer to the revised version.

Lines 33-35 could add reference: Howard, I. S., Wolpert, D. M., & Franklin, D. W. (2013). The effect of contextual cues on the encoding of motor memories. Journal of neurophysiology, 109(10), 2632-2644.

We thank the reviewer for this reference. We have added this to the introduction, where suggested, as well as in the Discussion, when writing about the reliability of different types of contextual cues.

Lines 61-63, “Heald et al. (2021) formalized context inference as a process that operates independently from motor learning, but is informed by it, establishing a loop whereby context inference also informs motor learning.” but is informed by it - this is unclear

We have rephrased this sentence to clarify the meaning. The new sentence (in lines 62-63) reads: "Heald et al. (2021) formalized context inference as a process that operates independently from motor learning, but both informs and is informed by it."

10. Figure 1. How are motor commands “observable”?

We agree that this is confusing. In this case, by 'observable' we meant something to which the model and the brain have access to during movement and adaptation. It is not meant as observable by experimenters. We have added a clarification to the caption of Figure 2.

TODO Line 160 add “s” to provide

We thank the reviewer for this comment. However, as we refer not to the field of study but to the 'forces' causing the phenomena, we believe that the plural matches the usage of dynamics in this context.

Line 176-177 “in which the mechanical arm forces the participant to make straight-line movements” This is incorrect. The robot is programmed with spring and damping coefficients to match the force exerted by the participant and make it appear as though they are making a perfect trajectory from start position to target (i.e., the elimination of the visual error).

We agree with the reviewer that the statement was confusing. We have now rephrased this line, which can now be found in lines 246-248. Additionally, we provide a better explanation for EC trials in both mechanical arm and visuomotor rotation experiments in the new section "Experimental designs", in the paragraph in lines 132-143.

Lines 182-183 “With repeated contexts (e.g. Oh & Schweighofer, 2019), an experiment can be described as O1. A1.O2”. This is unclear.

We rephrased this sentence and added more clarification. The paragraph can be found in lines 249-255.

Line 186 “without having to re-learn it.” They are learning in the second session of A, it is just that they are learning at a faster rate.

We re-formulated and clarified that we make a distinction between fast learning on repeated exposure and ‘switching’, as it is often referred to. Switching can be seen, for example, in the experiments by Kim (2015), where learning "picks up where it left off" when a previous adaptation is encountered, and in the experiments by Oh and Schweighofer (2019).

We have added these clarifications to the manuscript, see the paragraphs in lines 273-274 and 286-289.

Line 192. In the baseline condition there is nothing to learn (i.e., there is not perturbation). In this case there is a washout or decay of the learning of A.

We agree with the reviewer. However, because the baseline context holds no special place in the model (outside of having been learned for long enough that any new learning occurs very slowly, or not at all during an experiment), we wanted to emphasize that there was no need to learn anything in these transitions in preparation for the results.

Line 195 and 199 replace “are” with “is”

Usages of "savings" in published work on motor adaptation with grammar that allows for a distinction between plural and singular are mixed; for example, Oh and Schweighofer (2019) chose plural ("Savings were dominant in the large perturbation conditions. . . "), while Herzfeld et al. (2014) chose singular ("Savings refers to the. . . "). Given the ambiguity in usage, we prefer to follow the accepted dictionary entries, which all use the plural and are consistent across the dictionaries by Merriam-Webster, Oxford and Collins. We found a single use of 'savings' in singular in our manuscript and have changed it to plural, to be consistent with the rest.

Did the simulated data represent the same number of subjects reported in the compared studies? This is unclear in the reading.

We thank the reviewer for this comment. All simulations with a direct experimental counterpart were done with the same number of runs as there are participants.

For clarification, alongside short explanations for the experimental designs we discuss (new figure 1), we have added notes on the number of participants (per group) used by the experiments cited (in their corresponding figure captions), as well as the number of simulations run with our model.

Can the authors explain why their model predicts no savings upon the 4th presentation of the perturbation when the behavioural data to the right clearly shows savings. (From figure 2C). The learning rate of the model clearly lags behind the data.

Certainly, this is an important point. The observed difference between blocks in the simulations in Figure 3 is rather random, and depends on the sampled observations and motor noise in the model at the time of running the simulations.
To explain, the model does not have any direct mechanism for savings in terms of a speed-up of learning in blocks to which a participant had previously been exposed. Instead, this phenomenon is modeled as a consequence of the process of context inference, which is slower for smaller visuomotor rotations. In the presence of unambiguous and reliable contextual queues, immediate switches are observed between previously-encountered blocks, even if learning has not been completed, as in Figure 3A, while an apparent accelerated re-learning is observed when the inference over switches between context is slower, as in Figure 3C.
We have added this discussion to the "Cue- and sensory feedback uncertainty affects switching behavior" section, lines 286-289 and 337-340.

Lines 407 - 413 There is no mention of the decay happening in error clamps. "Instead, participants slowly reduce their adaptation, often displaying spontaneous recovery" FIrst you get rebound and then you get decay . . . this statement is misleading.

We have rephrased this to add that spontaneous recovery happens before decay. This can be found in lines 493-494.

Experiments often interleave error clamp trials within the adaptation blocks. Can your sCOIN model account for the changes in behaviour during these trials, given that participants are often unaware they even occur?

Yes, we believe the model can account for this phenomenon. As can be seen in Figure 5C, the model needs many EC trials to fully infer that the previous context is no longer active. Looking at Figure 5C, one can see that the effect of one to three EC trials in the middle of an adaptation block would have very little effect in behavior, mirroring human behavior in these test EC trials during experiments.

Furthermore, the model would also issue motor commands consistent with the adaptation block, which could be used to "measure" its response (the counteracting force) while still having it observe zero error.
Briefly connecting this discussion with Scheidt et al. (2000) reference in a comment below: the behavior of the model during interleaved EC-Null blocks, as done by Scheidt et al. for their control group, would trivially mimic the behavior they observed in their participants: on the first null trial, context inference would quickly infer the baseline context and behavior would quickly become consistent with it, as happened in our simulations in Figure 2A.
To show this more clearly, we have now incorporated random EC trials within the adaptation blocks of the Vaswani et al. (2013) simulations (figure 5), which we had omitted before because in the experiments they had been included randomly, in sequences we had no information available to reproduce. As can be seen now in Figure 5, their inclusion does not significantly affect context inference, and its effects on simulated behavior (Figure 5A) are negligible.

It would also be interesting to see not only the simulated data from the sCOIN model but also the COIN model to demonstrate where the COIN model fails to predict the data while the sCOIN improves the prediction.

We concur with the reviewer that this would be interesting. However, it was not our intention to challenge the COIN model, nor to claim that the simplified version used here is better or different in any way other than its simplicity, as we suggest by the name. Indeed, as we mention in the Discussion and Methods section, the sCOIN model strips away some functionality from the COIN model.
Importantly, the simplifications made for this model do not alter the general behavior of the model, nor do they impact its ability to explain the phenomena discussed in the manuscript. These simplifications allow for a more intuitive interpretation of the model parameters in terms of learning rates and the stability of learned adaptations, compared to the COIN model, incorporating perceptual and motor noise, as was discussed at the end of the Methods section.
Given that the components removed from COIN to make sCOIN do not affect the mechanisms discussed in this manuscript, we expect the results to be similar, if not identical, if using the COIN model, only having to set different parameters to match the observed behavior.

We have added to the Introduction an emphasis on our intentions regarding the modeling (lines 68-72).

Overall there lacks a clear and direct comparison (statistically between simulated outcomes and behavioural data). The comparisons are of a more qualitative nature. The authors could provide a regression metric.

We thank the reviewer for this question. There were three reasons why we performed qualitative comparisons with selected parameter values. Firstly, the key point of the manuscript is to show that the context-inference approach shown here provides a unifying explanation for a host of phenomena discussed in this manuscript, as well as in Heald (2021) and Oh (2019). Using the current approach shows that the adaptability of the model supports our conclusions.
Secondly, our intention was to show that the concept of context inference itself is responsible for the behavioral phenomena, and not a specific mathematical implementation of the model, or the fitting process. Although showing a fitting process would be clearly useful for future experimental applications, the fitting itself would not have enhanced the conclusions reached in this manuscript.
Thirdly, we believe that it would be best to design new experiments specifically to test the predictions laid out in the Discussion section and design the fitting procedure accordingly. Under these circumstances, a qualitative comparison between the behavior predicted by the model and that of participants can be done directly. We will do this in future work.
We have added this discussion to the Discussion section of the manuscript (subsection called "Future work", lines 603-608).

413-417 Participants are often unaware of the presence of error clamps. How can the authors account for this finding in relation to the literature? I.e., Scheidt, R. A., Reinkensmeyer, D. J., Conditt, M. A., Rymer, W. Z., & Mussa-Ivaldi, F. A. (2000). Persistence of motor adaptation during constrained, multi-joint, arm movements. Journal of neurophysiology, 84(2), 853-862.

This is an interesting point. We would argue that the marked difference between participants in the control and those in the test group (as seen in their Figure 5) indicates that "being aware" is a continuous dimension; the presence of null trials in the channel-null block near the end provides precise

feedback of the change (i.e. that they are no longer in the adaptation block) in the form of large kinematic error. Conversely, participants in the test group have no access to these kinematic errors. This, however, does not mean that they are fully unaware that a change has occurred, as we now eplain in lines 423-426, as well as the existing arguments in the paragraph starting in line 396.
As can be seen in their figure 5, the peak force varies wildly from trial to trial for both groups, even towards the end of the adaptation block (at which point they have already learned the adaptation fairly well, as the authors remarked). During adaptation or null trials, such variation would lead to big kinematic errors. The absence of these errors is in itself a great source of information which, as it happens in context inference in our model, tells the participant that something has changed.
Scheidt et al. briefly tested whether vision was the driving force with a single participant for whom the view to the hand and the cursor on the screen was blocked during the EC trials. The authors concluded that visually observed kinematic error was not the driving force behind the loss of adaptation. This, however, does not preclude other sources of information, multiple of which are mentioned by Scheidt et al. (e.g. "Specifically, the slow loss of adaptation of our test subjects was likely driven by Golgi tendon organs and/or hand mechanoreceptors…"). While we do not explicitly model these sources of information (instead relying on visually-observed kinematic errors as the main driving force behind adaptation and switching), they fit right in the context inference account and would likely be part of the multisensory-like integration mentioned in the Discussion section of this manuscript.

Rather than separate presentations of simulated model output and experimental data, these two learning curves could be overlaid in contrasting colors to highlight their similarities or differences

We thank the reviewer for this suggestion. We tried doing this, but because of the amount of information presented in figure 3 (previously figure 2), stacking experimental data and simulated data in one panel leads to a visually unclear image. To show this direct comparison, we created a supplementary figure S1, in which the experimental and simulated data are stacked without any error bars, and in a bigger format. In the manuscript, we refer to this figure in line 319.

Typos and small corrections

The following typos and other erroneous details were corrected:

1. Lines 40-41 add "upon re-exposure to the same perturbation". The ability to recall previously learned skills upon re-exposure to the same perturbation,
2. Line 47 - models don't do the adapting. "Whereby after each iteration, the change in motor commands is related to the measure of error."
3. Line 47 The most well-studied models are ..."
4. Lines 49-50 from "but Bayesian accounts have also been presented, providing an alternative explanation for savings and quick de-adaptation in the form of switching between forward models" to "while Bayesian models have been shown to provide an alternative explanation for savings and quick de-adaptation in the form of switching between forward models."
5. Line 66 - in this work we show
6. Line 70 - remove "so-called"
7. Line 104 - add "s" to component
8. Lines 137-138. The robot motors exert a force on the handle of the robot, held by the participant. It does not exert force on the participant's hand.
9. Lines 154-157. More clearly stated as the output of the simulated data qualitatively matches the behavioural data observed in ..... <list of studies>
10. Line 348, had begun or began
