# Supplementary material for: The effects of probabilistic context inference on motor adaptation
Source: PLoS One. 2023 Jul 3;18(7):e0286749. doi: 10.1371/journal.pone.0286749 (PMC10317241; doi:10.1371/journal.pone.0286749)
Supplement: Attachment — Submitted filename: reviewers_comments_second_round.pdf [file pone.0286749.s004.pdf]

Manuscript changes

Dario Cuevas, Stefan Kiebel

May 11, 2023

Response to reviewers' comments

Reviewer 2

We appreciate the reviewer's suggestions and have addressed them all below. In our responses below, all line numbers refer to the new manuscript; however, the reviewers' comments refer to the lines in the previous submission.

0.1 Citation needed, line 36

The statement was originally meant as an every-day example of motor adaptation; we have now changed it to a reference to known experiments in the literature and added example references.

0.2 Citation needed, line 272

We have added a citation to Ethier et al 2008 for an example of quick de-adaptation.

0.3 Add legends, figure 3

We have added legends for the contexts in the first two columns of the figure, as well as added a clarification to the O-A-B nomenclature in the caption.

0.4 Add legends, figure 5C

We have added legends for the contexts in figure 5C. In addition, we updated the panels to include the randomly-placed EC trials throughout the adaptation blocks, as stated in the main text.

0.5 Might be good to add a vertical line at 0, figure 5

For clarification, we added the vertical line at 0, as suggested by the reviewer, as well as aligned the x-axis to those of plots 5A and 5B, to improve clarity.
